# Transmission of SARS-CoV-2 After COVID-19 Screening and Mitigation Measures for Primary School Children Attending School in Liège, Belgium

**DOI:** 10.1001/jamanetworkopen.2021.28757

**Published:** 2021-10-12

**Authors:** Christelle Meuris, Cécile Kremer, Anton Geerinck, Medea Locquet, Olivier Bruyère, Justine Defêche, Cécile Meex, Marie-Pierre Hayette, Loic Duchene, Patricia Dellot, Samira Azarzar, Nicole Maréchal, Anne-Sophie Sauvage, Frederic Frippiat, Jean-Baptiste Giot, Philippe Léonard, Karine Fombellida, Michel Moutschen, Keith Durkin, Maria Artesi, Vincent Bours, Christel Faes, Niel Hens, Gilles Darcis

**Affiliations:** 1 Department of Infectious Diseases, Liège University Hospital, Liège, Belgium; 2 I-BioStat, Data Science Institute, Hasselt University, Hasselt, Belgium; 3 World Health Organization Collaborating Center for Public Health Aspects of Musculo-Skeletal Health and Ageing, Division of Public Health, Epidemiology and Health Economics, University of Liège, Liège, Belgium; 4 Department of Clinical Microbiology, University of Liège, Liège, Belgium; 5 Department of Human Genetics, Centre Hospitalier Universitaire Liège, Medical Genomics, Groupe Interdisciplinaire et Génoprotéomique Appliquée Research Center, University of Liège, Liège, Belgium; 6 Centre for Health Economics Research and Modelling Infectious Diseases, Vaccine and Infectious Disease Institute, University of Antwerp, Antwerp, Belgium

## Abstract

**Importance:**

Recent data suggest a relatively low incidence of COVID-19 among children. The possible role that children attending primary school may play in the transmission of SARS-CoV-2 remains poorly understood.

**Objective:**

To gain a better understanding of the possible role of children in the transmission of SARS-CoV-2.

**Design, Setting, and Participants:**

This prospective cohort study was conducted from September 21 to December 31, 2020, in a primary school in Liège, Belgium, among a volunteer sample of 181 children, parents, and school employees.

**Exposures:**

Participants were tested for SARS-CoV-2 infection once a week for 15 weeks through throat washing, performed with 5 mL of saline and collected in a sterile tube after approximately 30 seconds of gargling. Quantitative reverse transcription–polymerase chain reaction was performed to detect SARS-CoV-2 infection.

**Main Outcomes and Measures:**

In case of test positivity, participants were asked to complete a questionnaire aimed at determining the timing of symptom onset and symptom duration. SARS-CoV-2 genetic sequencing was also performed. Confirmed cases were linked based on available information on known contacts and viral sequences.

**Results:**

A total of 181 individuals participated in this study, including 63 children (34 girls [54.0%]; mean [SD] age, 8.6 [1.9] years [range, 5-13 years]) and 118 adults (75 women [63.6%]; mean [SD] age, 42.5 [5.7] years [range, 30-59 years]). Forty-five individuals (24.9%) tested positive: 13 children (20.6%; 95% CI, 10.6%-30.6%) and 32 adults (27.1%; 95% CI, 19.1%-35.7%) (*P* = .34). Children were more often asymptomatic compared with adults (6 [46.2%; 95% CI, 19.1%-73.3%] vs 4 of 31 [12.9%; 95% CI, 1.3%-24.5%]; *P* = .04). The median duration of symptoms was shorter in children than in adults (0.00 days [IQR, 0.00-1.00 days] vs 15.00 days [IQR, 7.00-22.00 days]). A reconstruction of the outbreak revealed that most transmission events occurred between teachers and between children within the school. Of the observed household transmission events, most seemed to have originated from a child or teacher who acquired the infection at school.

**Conclusions and Relevance:**

Despite the implementation of several mitigation measures, the incidence of COVID-19 among children attending primary school in this study was comparable to that observed among teachers and parents. Transmission tree reconstruction suggests that most transmission events originated from within the school. Additional measures should be considered to reduce the transmission of SARS-CoV-2 at school, including intensified testing.

## Key Points

**Question:**

What is the possible role of children in SARS-CoV-2 transmission?

**Findings:**

This cohort study including 63 children and 118 adults found no significant difference between the number of children and the number of adults testing positive for SARS-CoV-2 infection during the study period; children were asymptomatic significantly more often compared with adults (46% vs 13%). In addition, a reconstruction of the outbreak showed that most transmission events originated from within the school.

**Meaning:**

These results suggest that children may play a larger role in the transmission of SARS-CoV-2 than previously assumed.

## Teaser

This cohort study uses data from a primary school in Belgium to examine the possible role of children in the transmission of SARS-CoV-2.

## Introduction

The clinical manifestations of SARS-CoV-2 infection in children are relatively benign, with 90% remaining completely asymptomatic or having mild-to-moderate disease.^1^ Nevertheless, the implications of having children who are asymptomatic but potentially infectious in the community are of concern. Most countries around the world have thus implemented a range of community containment strategies to prevent the transmission of SARS-CoV-2, including school closures. Restrictions on activities have been imposed in schools that have remained open regardless of the evidence for the effectiveness of school closures and other school social distancing measures, which have mainly been derived from past influenza outbreaks for which children played a role in viral transmission.^2^ Using these data, school closures were implemented almost ubiquitously.^3^

School closures may have several negative effects at the individual and community levels. In addition to the possible negative effects on learning, there may be a decrease in physical activity as well as a variety of possible negative effects on mental health and well-being.^4^

A more comprehensive understanding of the susceptibility of children to SARS-CoV-2 infection and their possible role in transmission is needed to help define strategies aimed at addressing the COVID-19 pandemic while preserving families’ well-being.^2^ To date, most evidence comes from widespread community testing. In Iceland, children younger than 10 years of age were less likely to receive a positive test result than older individuals who received targeted testing; in the population screening, no child younger than 10 years of age had a positive test result.^5^ Data from Italy and Japan tend to confirm the low rates of infection among children.^6,7^ Zhu and colleagues^8^ recently showed that, in household transmission clusters of SARS-CoV-2, the index case is unlikely to be a child. Ismail and colleagues^9^ estimated the rate of SARS-CoV-2 infection and outbreaks among staff members and students in educational settings during the summer half-term (June and July 2020) in England. The staff members had a higher incidence of infection than did the students, and most of the cases of infection associated with the outbreaks were staff members. Li and colleagues^10^ recently reported that the proportion of children among all patients with confirmed COVID-19 estimated for 29 countries varied from 0.3% to 13.8%. In Australia, SARS-CoV-2 transmission rates were low (1.2%) in educational settings during the first epidemic wave.^11^

Overall, much of the recent data has suggested that children do not play a significant role in the transmission of SARS-CoV-2.^10^ However, the available evidence for quantifying the extent to which children may contribute to overall transmission is limited. The possible role that children play in the transmission of SARS-CoV-2 and the rate of infection among children may be underestimated because children are more often asymptomatic.^12^ Samples collected by nasopharyngeal swab may increase the percentage of false-negative test results. This sampling technique is not comfortable, particularly for less-compliant patients, such as young children.^13^

We present a prospective study in a primary school located in Liège, Belgium, involving children, parents, and teachers, with the objective of better understanding the possible role that children play in the pandemic. Participants were tested once a week for SARS-CoV-2 infection through throat washing, a nontraumatic, sensitive technique that alleviates the demand for supplies of swabs and personal protective equipment.^14^ Sequencing was systematically performed to provide a rigorous molecular-based analysis of COVID-19 clusters. This study took place during the second COVID-19 wave in Belgium in October and November 2020.

## Methods

### Participant Enrollment

We conducted prospective surveillance of SARS-CoV-2 throat carriage among children, parents, and employees in a single elementary school in Liège, Belgium, from September 21 (calendar week 39) to December 31, 2020. Data were analyzed after 15 weeks. The protocol was approved by the ethics committee of Liège University Hospital, the school directors, and the local authorities. Employees as well as parents of all primary school children were contacted by email. If they were interested in participating in the study, they received explanations and provided informed consent. The investigator was available for questions by mail and telephone throughout the study period. All adults provided written informed consent, and the children signed an adapted informed consent form. Mitigation measures implemented in the school evolved during the course of the study, according to national guidelines (see eMethods in the Supplement for a detailed description). This study followed the Strengthening the Reporting of Observational Studies in Epidemiology (STROBE) reporting guideline.

### Sample Collection and SARS-CoV-2 Detection

Screening for SARS-CoV-2 was performed once a week for all study participants through throat washing. Participants were asked to collect specimens in the morning before eating, drinking, or teeth brushing. Throat washing was performed with 5 mL of saline and collected in a sterile tube after approximately 30 seconds of gargling. This technique has been shown to be sensitive for the detection of SARS-CoV-2.^14^ Quantitative reverse transcription–polymerase chain reaction was performed to detect SARS-CoV-2 infection. SARS-CoV-2 sequencing was performed as described by Freed and colleagues^15^ (eMethods in the Supplement).

If results were positive, the participant was called by the investigators and isolated to limit the possible spread of the virus. Family members were isolated as well, and advice was given to prevent transmission within the household. The duration of isolation was initially 7 days, but national guidelines for quarantine changed during the study to 14 days, then 7 days, and finally 10 days.

 Participants who tested positive were asked to complete a questionnaire aimed at determining the timing of symptom onset and symptom duration.

### Statistical Analysis

Continuous variables were reported as mean (SD) values or as median (IQR) values depending on the normality of their distribution. Nominal variables were reported as absolute and relative frequencies (numbers and percentages). The significance of the difference in the mean values between the 2 groups was assessed using *t* tests (parametric) or Mann-Whitney tests (nonparametric). For nominal values between 2 groups, we calculated *P* values using Pearson χ^2^ tests or Fisher exact tests if conditions were not satisfied. The difference in the infection rates between adults and children was compared using mixed-effects logistic regression with random intercepts for classroom and household, accounting for the clustering of individuals. A mixed-effects Cox proportional hazards regression model was used to investigate whether the time to testing positive for SARS-CoV-2 was different between adults and children, quantified as a hazard ratio with 95% CI. Kaplan-Meier survival curves were constructed to visually demonstrate the infection distribution between adults and children over time. All *P* values were from 2-sided tests and results were deemed statistically significant at *P* ≤ .05. Analyses were performed with IBM SPSS for Windows, version 27.0.0.0 (IBM Corp) and R, version 3.6.3 (R Group for Statistical Computing).

### Reconstruction of Outbreak

A previously developed method was used for estimating the generation interval (ie, the time between 2 infections) based on symptom-onset data and reconstruction of the most likely transmission tree.^16^ In brief, this method assumes that the incubation period of the infector is independent of the generation interval, such that the serial interval (ie, the time between symptom onset of a new case and symptom onset of its infector) can be seen as a convolution of the generation interval and the difference between the incubation period of the infector and the incubation period of the infectee. Here we constrained the serial interval to be larger than −5 days.^17^ The present study reflects a partially observed outbreak; not all classmates and household members of an individual who tested positive for SARS-CoV-2 were included in the study. Inference based only on the observed cases may lead to an overestimation of the generation and serial intervals, if cases between 2 infections are missed.^18^ We therefore extended the method to account for unobserved (intermediate) cases by adjusting the likelihood (eMethods in the Supplement). Individuals confirmed as being a case were linked based on available information on known contacts and viral sequences. Detailed assumptions can be found in the eMethods in the Supplement. Parameter estimation was performed using Markov chain Monte Carlo methods.

## Results

### Characteristics of the Study Population

A total of 181 individuals participated in this study. The sample consisted of 63 children (34.8%), 82 parents (45.3%) of these children, 17 school employees (9.4%), 15 teachers (8.3%), and 4 participants (2.2%) who were both teacher and parent of a child included in the study (Table 1). Children were aged 5 to 13 years (mean [SD] age, 8.6 [1.9] years). Adults were aged 30 to 59 years (mean [SD] age, 42.5 [5.7] years). We characterized the link between children and parents by categorizing them in households. The 63 children and 83 parents comprised 47 households, including 16 sibling pairs. Included children and teachers were part of 13 class groups at the primary school level as well as kindergarten (eTable 1 in the Supplement).

**Table 1.** Characteristics of the Study Population by Group

Characteristic	Participants, No. (%)					
	Children (n = 63)	Adults (n = 118)	Parents (n = 82)	School employees (n = 17)	Teachers (n = 15)	Parents-teachers (n = 4)
Age, mean (SD) [range], y	8.6 (1.9) [5-13]	42.5 (5.7) [30-59]	42.8 (5.0) [33-59]	40.4 (5.5) [31-49]	43.9 (8.9) [30-57]	41.0 (2.8) [37-43]
Sex						
Female	34 (54.0)	75 (63.6)	44 (53.7)	14 (82.4)	13 (86.7)	4 (100)
Male	29 (46.0)	43 (36.4)	38 (46.3)	3 (17.6)	2 (13.3)	0

A total of 2015 samples were collected during the study, with a median number of 155 (IQR, 126-163) analyzed samples per week. The median number of samples per patient was 12 (IQR, 10-13), reflecting high adherence to the study protocol.

### Incidence of COVID-19 in the Study Population

During the entire study duration, 45 individuals (24.9%) tested positive for SARS-CoV-2: 13 children (20.6%; 95% CI, 10.6%-30.6%) and 32 adults (27.1%; 95% CI, 19.1%-35.7%; *P* = .34) (Table 2). When accounting for clustering of individuals in classrooms and households, there was no significant difference in infection rates between children and adults (odds ratio, 0.58 [95% CI, 0.22-1.41]; *P* = .25). The intraclass correlation was 0.39 for household and 0.15 for classroom. There was also no difference between specific adult groups regarding the prevalence of COVID-19 (25.6% for parents [21 of 82], 40.0% for teachers [6 of 15], 17.6% for school employees [3 of 17], and 50.0% for teacher-parents [2 of 4]; *P* = .56) (Table 2).

**Table 2.** Cumulative Incidence of COVID-19 in the Sample

Population	Positive/total sample (%)
Total sample	45/181 (24.9)
Children	13/63 (20.6)
Adults	32/118 (27.1)
Parents	21/82 (25.6)
Teachers	6/15 (40.0)
Employees	3/17 (17.6)
Teacher-parents	2/4 (50.0)

**Figure 1.**
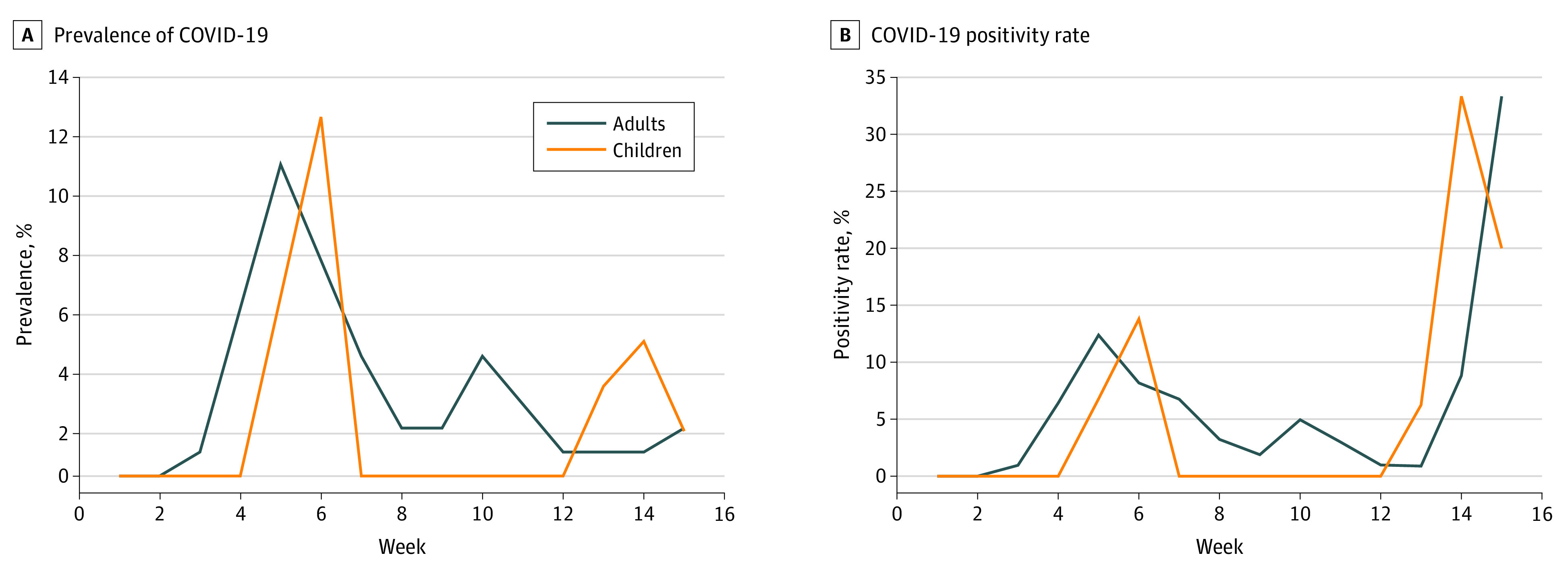
Prevalence of COVID-19 (A) and Positivity Rate (B) Among Children and Adults

Figure 1A represents the weekly prevalence among children and adults, and Figure 1B shows the positivity rate over time. Both groups experienced a concomitant infection peak around week 5 and a second smaller infection peak later in the study. eFigure 1 in the Supplement shows the Kaplan-Meier curves visualizing time to a positive test result for both groups, with 95% CIs. We investigated whether the time to testing positive for SARS-CoV-2 was different between adults and children using a mixed-effects Cox proportional hazards regression model to account for clustering of individuals in households and in classrooms. Results showed that children had a hazard ratio of 0.59 (95% CI, 0.30-1.18; *P* = .14), indicating no statistically significant difference in the time to testing positive for SARS-CoV-2 between children and adults. The intraclass correlation was 0.30 for household and 0.03 for classroom.

### Symptoms Experienced by SARS-CoV-2–Positive Participants

Data on the symptoms experienced by infected individuals were available for 44 of 45 participants. Thirty-four individuals were symptomatic, while 10 remained asymptomatic (eTable 2 in the Supplement). Children were more often asymptomatic compared with adults (6 [46.2%; 95% CI, 19.1%-73.3%] vs 4 of 31 [12.9%; 95% CI, 1.3%-24.5%]; *P* = .04). Data on symptom duration are shown in eTable 3 in the Supplement. The median length of symptoms was 8.50 days (IQR, 1-20 days) when both symptomatic and asymptomatic participants were included. The median duration of symptoms was shorter for children (0.00 days [IQR, 0.00-1.00 days]) than for adults (15.00 days [IQR, 7.00-22.00 days]). Two adults tested positive during 3 consecutive weeks, and 2 adults tested positive during 4 consecutive weeks (eTable 4 in the Supplement). Cycle threshold values at diagnosis did not differ between the 2 groups (children, 29.80 [95% CI, 28.31-31.10]; adults, 29.00 [95% CI, 23.49-35.56]; *P* = .51; eTable 5 in the Supplement).

### Reconstruction of Outbreak

eTable 7 in the Supplement shows the estimated parameters of the generation and serial interval distribution under several scenarios. Complete data used for outbreak reconstruction and a link to SARS-CoV-2 sequences are available in eTable 6 in the Supplement (includes codes to access to sequences on GISAID [Global Initiative on Sharing Avian Influenza Data] platform).^19^

**Figure 2.**
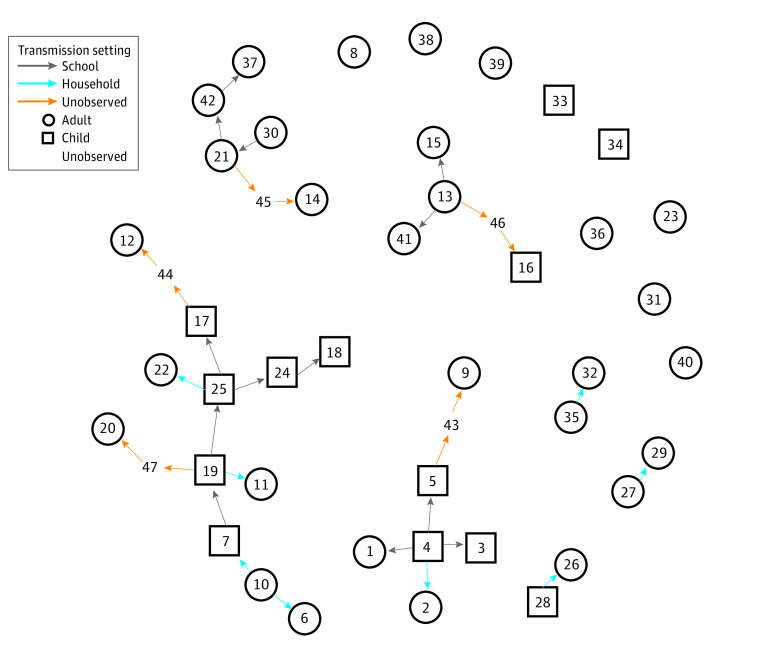
Most Likely Transmission Tree Under Baseline Scenario Arrows indicate likely transmission from case *i* to case *j* in school (gray arrow) or household (blue arrow). Transmission through an unobserved intermediate case is indicated by an orange arrow. The numbers indicate case identification numbers.

**Figure 3.**
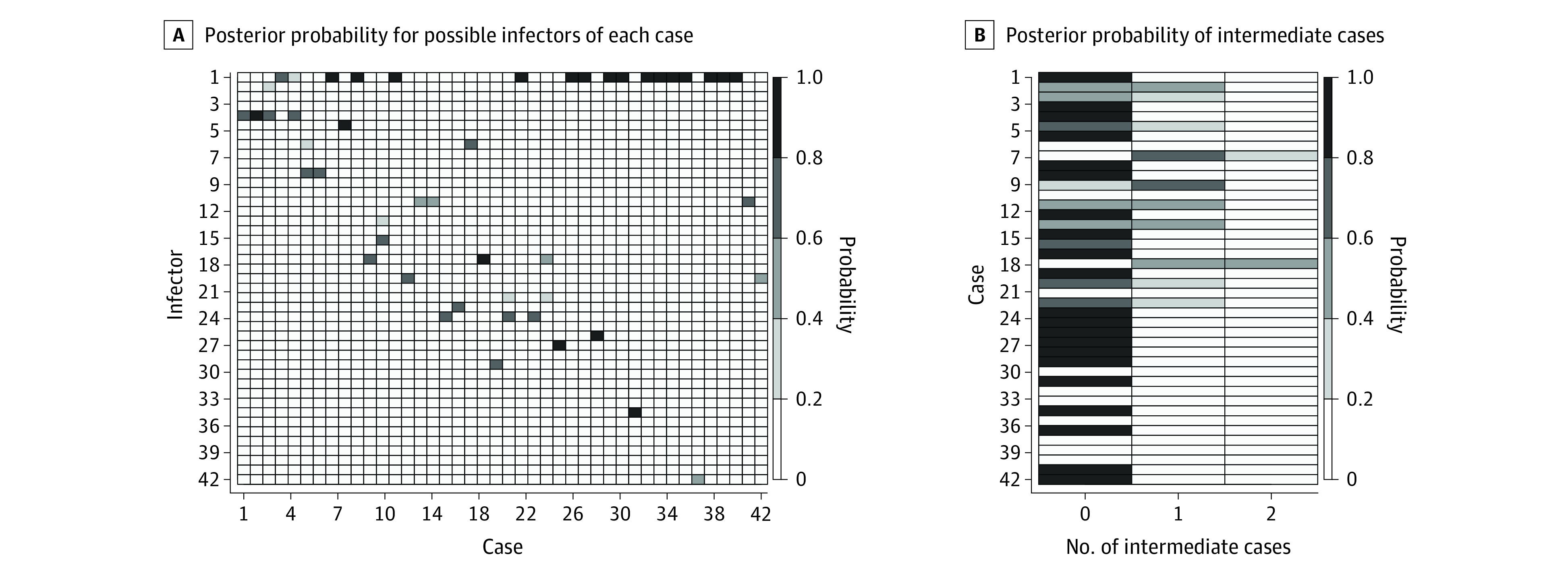
Posterior Probability for Possible Infectors of Each Case (A) and of Intermediate Cases (B) A, The x- and y-axes indicate case and infector identification numbers. B, The x-axis indicates the number of intermediate cases, and the y-axis indicates case identification numbers.

In our baseline scenario, we assumed that the probability of observation was 60%. The mean generation time was estimated to be 4.7 days (95% credible interval, 3.1-6.5 days) under this scenario. Figure 2 shows the most likely transmission tree corresponding to these estimates. An unobserved intermediate case was assigned when a case’s most likely value for κ under this most likely transmission tree was larger than 1. Under this scenario, 11 of the known infectors were adults, while 15 were children. We observed 8 adult-adult transmission pairs, 1 adult-child pair, 5 child-adult pairs, and 7 child-child pairs. Of the observed transmission events, 13 most likely occurred within the school, and 8 most likely occurred within households. For the 10 unobserved transmission events, we could assume that 4 occurred within the school (transmission chain from case 13 to case 46 to case 16, and transmission chain from case 21 to case 45 to case 14). To represent uncertainty in the most likely transmission tree, Figure 3A shows the posterior probability of all possible infectors for each case, where the first row indicates the probability of being an index case. Figure 3B shows the posterior probability of κ − 1 for each case, where κ − 1 represents the number of unobserved intermediate cases. We see, for instance, that case 9 has only 1 possible infector (case 5; Figure 3A) but also that there is most likely an unobserved intermediate case (Figure 3B).

We performed several sensitivity analyses to assess the robustness of these results. In scenarios 2 and 3, we fixed the probability of observation at 70% and 80%, respectively. Parameter estimates for the mean generation and serial interval were similar to those in the baseline scenario and slightly higher for the SDs (eTable 7 in the Supplement). Under scenario 2, upper bounds of the 95% credible interval for the SDs were higher, likely owing to suboptimal convergence (eFigure 4 in the Supplement) of the Markov chain Monte Carlo simulation. The most likely transmission trees obtained under scenarios 2 and 3 were the same as the transmission tree in the baseline scenario, although with a slight difference in the assignment of intermediate cases (eFigure 2 in the Supplement).

As an additional sensitivity analysis, we assumed that children could have been in contact only with their classmates instead of all other children within the school (scenario 4). For this scenario, adequate convergence (eFigure 4 in the Supplement) of the Markov chain Monte Carlo simulation was achieved only when assuming a probability of observation of 60%, requiring a careful interpretation of the results. The mean generation time was estimated to be 4.7 days (95% credible interval, 3.1-6.6 days), in line with our baseline scenario (eTable 7 in the Supplement). eFigure 3 in the Supplement shows the most likely transmission tree under this scenario. Among the observed transmission pairs, there are 10 adult-adult pairs, 3 adult-child pairs, 2 child-adult pairs, and 3 child-child pairs. Eleven of the observed transmission events were estimated to have most likely occurred within the school, and 7 within households. There were 10 unobserved transmission events, of which 8 were estimated to have occurred outside the school.

## Discussion

The education system has been severely affected by the COVID-19 pandemic. Understanding the possible role of children in the transmission of SARS-CoV-2 could assist in the development of operative ways to minimize the spread of SARS-CoV-2 while keeping educational institutions open.

Overall, previous studies mostly provided reassuring evidence regarding the possible role of children. In contrast, our study suggests that the incidence of COVID-19 among children may be higher than assumed. The measured cycle threshold values at diagnosis were also comparable between children and adults.

Because we had complete data regarding the timing of symptom onset and contact tracing, as well as SARS-CoV-2 sequences, we were able to reconstruct transmission trees. We observed that most of the transmissions occurred between teachers or employees and between children within the school, with spillover from children to their parents and teachers to their partners within the household. Although the most likely transmission tree may differ under different scenarios, the observed pattern of transmission was similar. Of the observed household transmission events in clusters consisting of more than 2 cases, most were from a child or teacher who acquired infection at school to their parent or partner within the household. This finding suggests that the observed transmission within households may have originated from someone who was infected within the school. These observations were made despite the implementation of several mitigation measures at school. However, mask wearing and physical distancing between children was not required, and extracurricular activities were allowed without any restrictions.

In this study, the possible role of children in the transmission of SARS-CoV-2 is likely owing to several specifics of the study design: (1) COVID-19 diagnosis was performed through a nontraumatic, sensitive technique well adapted to children; (2) testing was performed on a regular basis, independent of symptoms; and (3) the study took place during the second wave of the COVID-19 pandemic in Belgium, which significantly affected the region of Liège. National restrictions (mandatory teleworking, curfew, and closure of restaurants and bars) were implemented on October 19, 2020 (4 weeks after the study was initiated). Schools were closed on October 30, 2020, for 2 weeks. In addition, nonessential shops, contact professions, zoos, and holiday parks were closed on November 2, 2020 (6 weeks after the study was initiated).

### Limitations

This study has some limitations. In our method for outbreak reconstruction, ideally, both the incubation period and the generation time should be estimated from the same data.^20^ Because we did not have data available for estimating the incubation period in this study, we made the simplifying assumption that the incubation period of the infector was independent of the generation time. We further assumed that the generation time between case *i* and case *j* was independent of the generation time between case *j* and case *m*. Although this is plausible to assume in a fully observed outbreak, in the present study, the time interval between *i* and *m* was restricted to what we observed (plus or minus a few days) (eMethods in the Supplement). We did not include the possibility of contraction of the generation interval, which is a strong assumption, especially in households. In addition, as mentioned previously, these data were generated from earlier in the pandemic and do not account for variants that are now prevalent or for vaccination of adults.

Despite these limitations, several sensitivity analyses led to the same conclusion that the possible role of children in the transmission of SARS-CoV-2 may be higher than previously assumed. In the event that information on genetic clusters might have been discarded when constructing the possible transmission trees, the only additional link would be between 1 child (case 28) and all other children.

## Conclusions

Our study suggests that additional measures should be considered to reduce the transmission of SARS-CoV-2 at school. Mitigation measures of testing, contact tracing, and isolation should be strengthened so that schools can continue to stay open and children and staff are safe. The possible role of schools in the transmission of SARS-CoV-2 should also be included in the discussion regarding vaccination strategies (in particular, whether to vaccinate children).

## Notes

Supplement.
eMethods.
**eFigure 1.** Kaplan-Meier Curves **eFigure 2.** Most Likely Transmission Tree Under Scenario 3, Assuming an 80% Probability of Observation (as Compared With a 60% Probability of Observation Under the Baseline Scenario) **eFigure 3.** Most Likely Transmission Tree Under Scenario 4, Assuming Children Only Had Contact With Their Own Classmates Instead of all Children in the School, and a 60% Probability of Observation **eFigure 4.** Trace Plots to Assess MCMC Convergence **eTable 1.** Distribution of Children According to Class Groups **eTable 2.** Symptoms Experienced in Complete Sample **eTable 3.** Duration of Symptoms **eTable 4.** Duration of Positivity **eTable 5.** Comparison of Cycle Thresholds Between Children and Adults **eTable 6.** Complete Data Used for Outbreak Reconstruction **eTable 7.** Posterior Point Estimates for the Parameters of the Generation and Serial Interval Distribution
