# Supplementary material for: Transmission of SARS-CoV-2 After COVID-19 Screening and Mitigation Measures for Primary School Children Attending School in Liège, Belgium
Source: JAMA Netw Open. 2021 Oct 12;4(10):e2128757. doi: 10.1001/jamanetworkopen.2021.28757 (PMC8511974; doi:10.1001/jamanetworkopen.2021.28757)
Supplement: Supplement. — eMethods. eFigure 1. Kaplan-Meier Curves eFigure 2. Most Likely Transmission Tree Under Scenario 3, Assuming an 80% Probability of Observation (as Compared With a 60% Probability of Observation Under the Baseline Scenario) eFigure 3. Most Likely Transmission Tree Under Scenario 4, Assuming Children Only Had Contact With Their Own Classmates Instead of all Children in the School, and a 60% Probability of Observation eFigure 4. Trace Plots to Assess MCMC Convergence eTable 1. Distribution of Children According to Class Groups eTable 2. Symptoms Experienced in Complete Sample eTable 3. Duration of Symptoms eTable 4. Duration of Positivity eTable 5. Comparison of Cycle Thresholds Between Children and Adults eTable 6. Complete Data Used for Outbreak Reconstruction eTable 7. Posterior Point Estimates for the Parameters of the Generation and Serial Interval Distribution [file jamanetwopen-e2128757-s001.pdf]

Supplementary Online Content

Meuris C, Kremer C, Geerinck A, et al. Transmission of SARS-CoV-2 after COVID-19 screening and mitigation measures for primary school children attending school in Liège, Belgium. *JAMA Netw Open.* 2021;4(10):e2128757.
doi:10.1001/jamanetworkopen.2021.28757

eMethods.

eFigure 1. Kaplan-Meier Curves

eFigure 2. Most Likely Transmission Tree Under Scenario 3, Assuming an 80% Probability of Observation (as Compared With a 60% Probability of Observation Under the Baseline Scenario)

eFigure 3. Most Likely Transmission Tree Under Scenario 4, Assuming Children Only Had Contact With Their Own Classmates Instead of all Children in the School, and a 60% Probability of Observation

eFigure 4. Trace Plots to Assess MCMC Convergence

eTable 1. Distribution of Children According to Class Groups

eTable 2. Symptoms Experienced in Complete Sample

eTable 3. Duration of Symptoms

eTable 4. Duration of Positivity

eTable 5. Comparison of Cycle Thresholds Between Children and Adults

eTable 6. Complete Data Used for Outbreak Reconstruction

eTable 7. Posterior Point Estimates for the Parameters of the Generation and Serial Interval Distribution

This supplementary material has been provided by the authors to give readers additional information about their work.

eMethods.

Mitigation measures implemented at school

The standards implemented in the school in September were hand hygiene and facial mask wearing for adults (allowing breaks within the classroom, when seated at their desk). Mask wearing and social distance between children was never implemented within the classroom nor during the breaks. From 16 November, children had to eat inside the classroom and adults could take off their mask only outside while respecting 1.5m of distance.

During the course of the study, national guidelines recommended testing children over 6 years of age if a major symptom (cough, dysgeusia, anosmia or dyspnea) or two minor symptoms (including fever, diarrhea, rhinitis, myalgia, headache) were present. Testing for asymptomatic contacts was interrupted between October 21 and November 22 because of a lack of testing capacity. According to the national guidelines, classroom contacts between children or children and adults were classified as low risk contacts. Testing was not recommended in case of only one positive case within a classroom. Class closure and 10 days quarantine occurred if more than two cases were detected within a classroom.

SARS-CoV-2 sequencing

RNA was extracted from throat washing (300µl) via a Maxwell 48 device using the Maxwell RSC Viral TNA kit (Promega) with a viral inactivation step using Proteinase K, following the manufacturer's instructions. RNA was eluted in 50µL of RNAse free water. 3.3 µl of the eluted RNA was combined with 1.2 µl of SuperScript IV VILOTM Master Mix and 1.5 µl of H2O to carry out Reverse Transcription. This was incubated at 25°C for 10 min, 50°C for 10 min and 85°C for 5 min. PCR was carried out using Q5® High-Fidelity DNA Polymerase (NEB) and primers to obtain 1,200 bp amplicons as described by Freed and colleagues[15]. PCR conditions followed the recommendations in the sequencing protocol of the ARTIC Network. We multiplexed the samples following the manufacturer's recommendations using the Oxford Nanopore Native Barcoding Expansion kits 1-12, 13-24, and 96 in conjunction with Ligation Sequencing Kit 109 (Oxford Nanopore). We carried out sequencing on a Minion using R9.4.1 flow cells.

Adjusted likelihood

Suppose there is a transmission chain *j* -> *m* -> *i*, of which we only observe cases *i* and *j*. Denote X the estimate of the generation time that we would obtain without accounting for the unobserved case. This is then in reality the sum of two generation times, i.e. $X = X_1 + X_2$, with X_1 the generation time between *j* and *m*, and X_2 the generation time between *m* and *i*. Assuming X_1 and X_2 are independent, and follow the same distribution Gamma(α_k, β), then the convolution $X \sim$ Gamma($\Sigma\ \alpha_k$, β). Let κ_i be the

number of generations between case i and j. In addition, if π is the probability of observation, the probability of missing $\kappa_i - 1$ cases before observing case i is

$$p(\kappa_i \mid \pi) = \text{NB}(1 \mid \kappa_i - 1, \pi).$$

Adapting the likelihood from Ganyani et al. to account for these unobserved intermediate cases, we obtain[18]

$$L\left(\Theta, v(i)^{miss} | z_i, v(i)^{obs}, \pi, \kappa_i\right) = \prod_{i=2}^{n} \left(\left[\frac{1}{J} \sum_{j=1}^{J} f^{\kappa_i}\left(z_i - y_j | \Theta\right) \right] p(\kappa_i | \pi) \right)$$

where $f^{\kappa_i}(.)$ is the convolution of κ_i i.i.d. Gamma distributions, i.e. $X \sim Gamma\left(\sum_{k=1}^{\kappa_i} \alpha_k, \beta\right)$.

MCMC algorithm

The algorithm consists of two steps that are repeated until convergence: 1) update the transmission tree and corresponding values for κ based on the current parameter values, and 2) update the parameter values based on the currently accepted transmission tree and κ. For computational reasons, 10 000 possible trees were sampled before running the MCMC, and during step 1 above one of these trees was randomly selected (for details see below). We assume that both the generation interval and the incubation period follow a Gamma distribution. The incubation period distribution was fixed with a mean of 5.2 and standard deviation of 2.8 days[19]. Minimally informative uniform priors were used for the parameters of the generation interval distribution, which were updated using a random-walk Metropolis-Hastings algorithm with a uniform proposal distribution[20]. The posterior distribution was evaluated using 3 000 000 iterations of which the first 1 000 000 were discarded as burn-in. Thinning was applied by taking every 200th iteration. Posterior point estimates of the mean and standard deviation of the generation interval distribution are given by the 50th percentiles of the converged MCMC chain. Credible intervals (CrI) are given by the 2.5th and 97.5th percentiles. From all accepted trees, we visualize the most likely transmission tree (i.e. the one that is most often accepted by the MCMC algorithm). These analyses were performed using R software version 3.6.1.

Convergence of the MCMC was assessed using trace plots. As can be seen in Figure S4, adequate convergence is reached for the mean generation interval in most scenarios. For scenario 2, discarding the first 4000 iterations seen in the plot results in similar estimates as the ones reported (4.88 (95%CrI 3.43 – 6.58)). Convergence of the variance parameters is suboptimal in scenarios 2 and 3, but we believe this does not affect our conclusions and this nuisance parameter was not of main interest.

© 2021 Meuris C et al. *JAMA Network Open.*

Construction of possible transmission trees

Confirmed cases were linked based on available information on known contacts and viral sequences. Cases that reported a high-risk contact with someone outside of the study population were assumed to have been infected by those contacts and were set as index cases (i.e. not infected by someone from the study population). We assumed that children could have had contact with their household members, the teacher of their own class, and all other children in the school. Teachers were assumed to have had contact with each other, other school employees, children from their class, and with their own household members. Parents were assumed to only have had contact with other study participants if they were household members. Contact between two cases belonging to a different genetic cluster was excluded. Some cases may have no possible contacts based on the above assumptions, and hence were excluded from the analyses. Sensitivity analysis was performed assuming that, within the school, children only had contact with their teacher and children from their own class.

To speed up the MCMC, 10 000 unique possible transmission trees were sampled beforehand as follows:

1) Define possible infectors based on known contacts and sequencing information
2) Impute missing dates of symptom onset:
    a. Sample from from range [$\min(t_{v(i)})$-5 ; t_i^{lab}] where t_i^{lab} is the time of positive test, and $\min(t_{v(i)})$ is the earliest symptom onset time among contacts of case *i*
    b. If onset times are also missing for all contacts of a case, we set the lower bound of the range to the earliest onset time in the cluster -5 days
3) Constrain serial interval to ≥ -5 days and consequently change the list of possible infectors
4) Make sure every cluster has an assigned index case to avoid cycles in the network
5) Sample a transmission tree based on the list of possible infectors (with equal probabilities) taking into account the above constraints
6) Repeat step 2-5 to obtain 10 000 sampled trees

Values for the number of generations (κ) between two observed cases were sampled as follows:

- κ = 1 if observed serial interval shorter or equal to 5 days -> no intermediate case
- κ = 1 or 2 if observed serial interval between 6 and 12 days -> one intermediate case if κ = 2
- κ = 2 or 3 if observed serial interval longer than 12 days -> two intermediate cases if κ = 3

© 2021 Meuris C et al. *JAMA Network Open.*

eFigure 1. Kaplan-Meier Curves

The survival plot below shows the incidence of positive tests over time for two groups in the sample, the children and all adults taken together. When accounting for clustering of individuals in households and classrooms, the time to positive test was not different between adults and children (p=0.14).

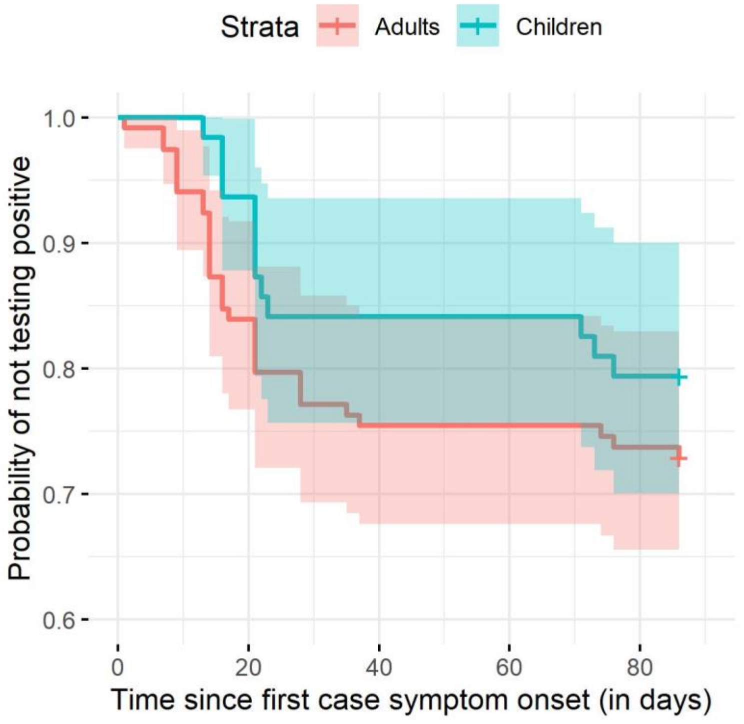

© 2021 Meuris C et al. *JAMA Network Open.*

eFigure 2. Most Likely Transmission Tree Under Scenario 3, Assuming an 80% Probability of Observation (as Compared With a 60% Probability of Observation Under the Baseline Scenario)

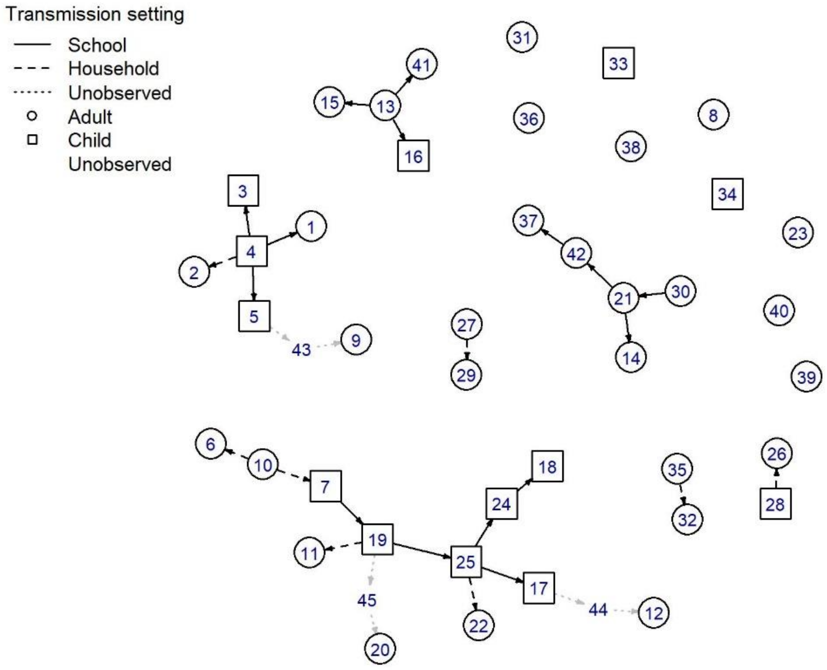

© 2021 Meuris C et al. *JAMA Network Open.*

eFigure 3. Most Likely Transmission Tree Under Scenario 4, Assuming Children Only Had Contact With Their Own Classmates Instead of all Children in the School, and a 60% Probability of Observation

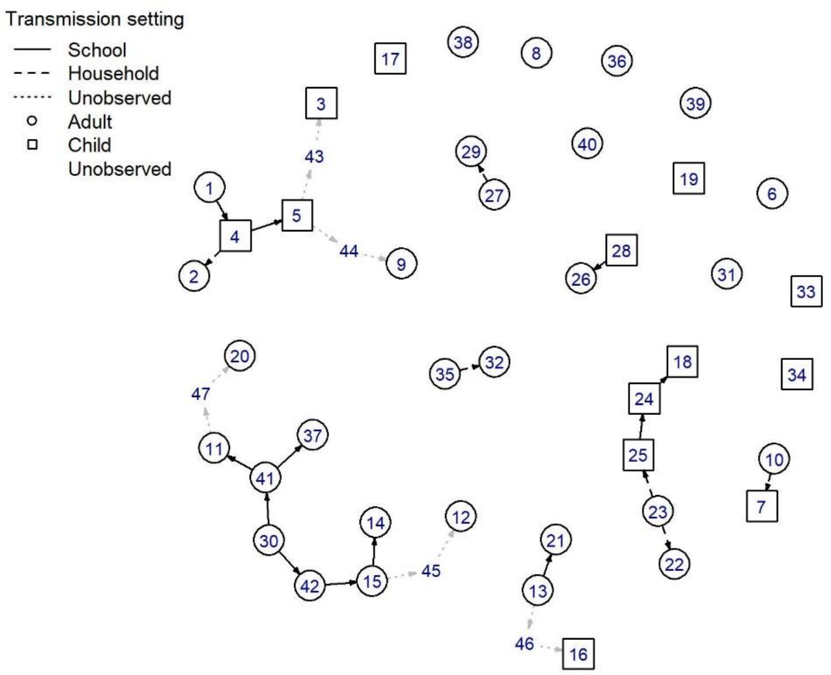

eFigure 4. Trace Plots to Assess MCMC Convergence (GI = generation interval).

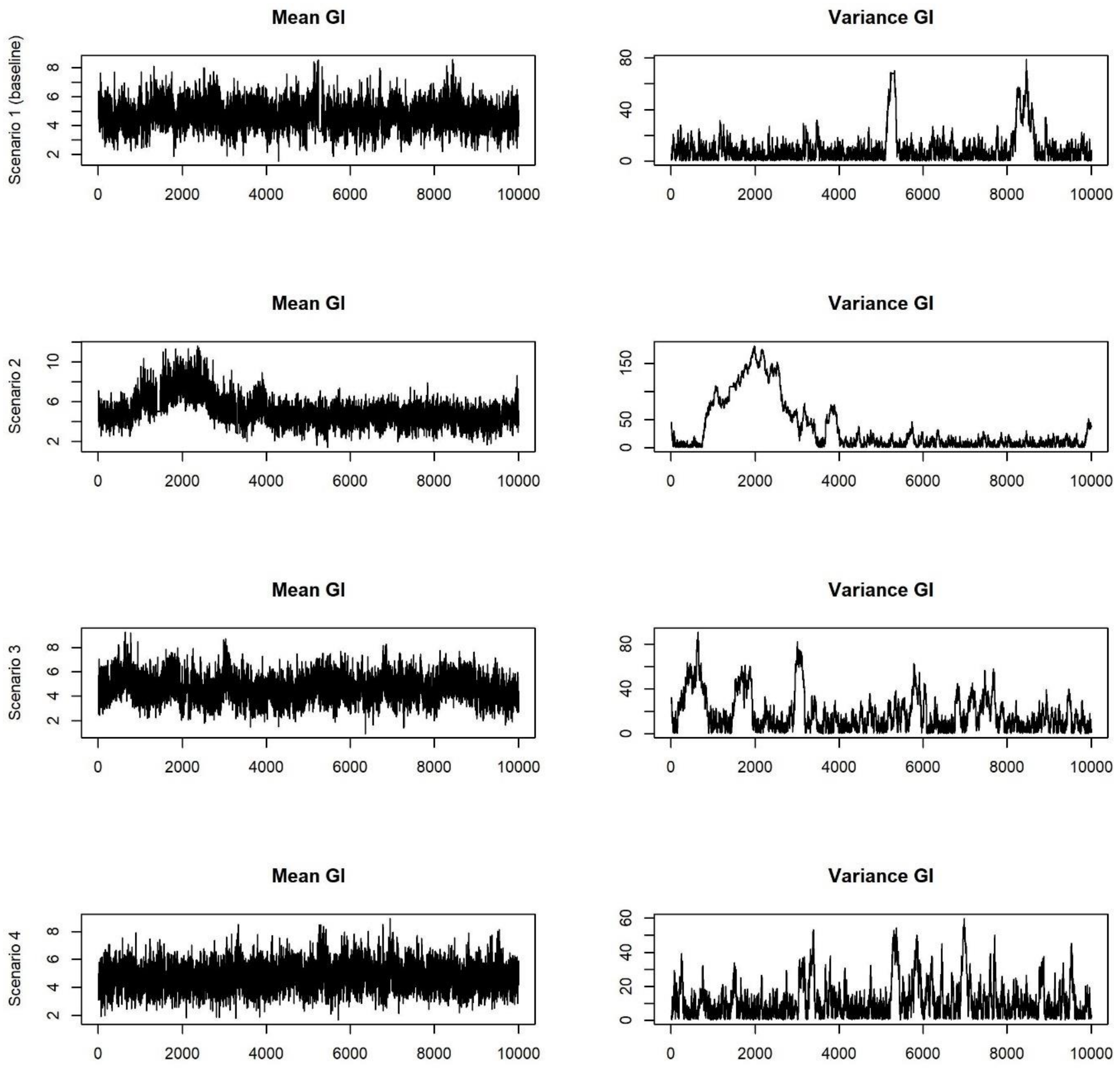

© 2021 Meuris C et al. *JAMA Network Open.*

eTable 1. Distribution of Children According to Class Groups

Table S1. Distribution of children according to class groups			
Class group	N	%	# Cases
Kindergarten	1	1.6	0
1A	4	6.3	1
1B	4	6.3	0
2A	6	9.5	3
2B	2	3.2	1
3A	5	7.9	1
3B	9	14.3	4
4A	4	6.3	0
4B	7	11.1	0
5A	7	11.1	0
5B	3	4.8	1
6A	4	6.3	1
6B	2	3.2	0
6C	4	6.3	1
Missing data	1	1.6	0

© 2021 Meuris C et al. *JAMA Network Open.*

eTable 2. Symptoms Experienced in Complete Sample

Table S2: Symptoms experienced in complete sample (n=45) – n(%)					
Code	Symptoms	Total (n=44)	Adults (n=31)	Children (n=13)	p-value
	**Asymptomatic**	10 (22.7%)	4 (12.9%)	6 (46.2%)	**0.043**
1	Fever	8 (18.2%)	6 (19.4%)	2 (15.4%)	1.000
2	Cough	8 (18.2%)	7 (22.6%)	1 (7.7%)	0.402
3	Ageusia	11 (25.0%)	10 (31.3%)	1 (7.7%)	0.132
4	Anosmia	18 (40.9%)	17 (54.8%)	1 (7.7%)	0.006
5	Headaches	15 (34.1%)	14 (45.2%)	1 (7.7%)	0.034
6	Myalgia	10 (22.7%)	10 (32.3%)	0 (0.0%)	0.021
7	Diarrhea	4 (9.1%)	4 (12.9%)	0 (0.0%)	0.302
8	Rhinorrhea	8 (18.2%)	7 (22.6%)	1 (7.7%)	0.402
10	Asthenia	21 (47.7%)	20 (64.5%)	1 (7.7%)	0.001
11	Anorexia	4 (9.1%)	4 (12.9%)	0 (0.0%)	0.302
12	Chest pain	7 (15.9%)	7 (22.6%)	0 (0.0%)	0.086
13	Heart palpitation	1 (2.3%)	1 (3.2%)	0 (0.0%)	1.000
14	Nausea	2 (4.5%)	1 (3.2%)	1 (7.7%)	0.508
15	Dyspnea	5 (11.4%)	5 (16.1%)	0 (0.0%)	0.301
16	Cutaneaous rash	2 (4.5%)	1 (3.2%)	1 (7.7%)	0.508
17	Abdominal pain	1 (2.3%)	1 (3.2%)	0 (0.0%)	1.000
Remark: Data missing for 1 participant, therefore % are calculated on a sample of 44/31/13. P-value from Fisher's exact test. One participant can experience multiple symptoms.					

© 2021 Meuris C et al. *JAMA Network Open.*

eTable 3. Duration of Symptoms

Table S3. Duration of symptoms		
Length (days)	n	%
0	10	23.8
1	4	9.5
4	2	4.8
7	4	9.5
8	1	2.4
9	1	2.4
11	1	2.4
14	2	4.8
15	5	11.9
20	2	4.8
21	2	4.8
22	1	2.4
24	1	2.4
25	1	2.4
33	1	2.4
40	2	4.8
41	1	2.4
56	1	2.4

© 2021 Meuris C et al. *JAMA Network Open.*

eTable 4. Duration of Positivity

Duration of positivity (in weeks)				
	Children (N=13)		Adults (N=32)	
	N	%	N	%
1	8	61.5	18	56.3
2	5	38.5	10	31.3
3	0	0.0	2	6.3
4	0	0.0	2	6.3

© 2021 Meuris C et al. *JAMA Network Open.*

eTable 5. Comparison of Cycle Thresholds Between Children and Adults

Table S5. Comparison of cycle thresholds between children and adult groups (n=45)			
	Children (n=13)	Adults (n=32)	P-value
Cycle threshold	29.80 (28.31-31.10)	29.00 (23.49-35.56)	0.51

© 2021 Meuris C et al. *JAMA Network Open.*

eTable 6. Complete Data Used for Outbreak Reconstruction

Link to SARS-CoV-2 sequences are available (see codes to access to sequences on GISAID (Global Initiative on Sharing Avian Influenza Data) platform https://www.gisaid.org/).

STUDY I+A1:S34D	Virus name	GISAID Accession ID	AGE	GENDER	SEQUENCING	SEQ. LINK?	HOUSEHOLD	DESCRIPTION	ID	TIME sympt c	TIME TEST	START ISOLA	END ISOLATI	CLUSTER1	CONTACTS1	TYPE.CT1	HR contact	CONTACTS2	Class1	Class2	Class3
0296322T	Belgium/ULG-11524/2020	EPI_ISL_925469	30	F	Cluster 1		63	teacher	1	69	70			Cluster 1	3, 4, 5	school		3, 4, 5	3B		
3236790W	Belgium/ULG-11527/2020	EPI_ISL_925471	43	F	Cluster 1		75	parent	2	76	72	72	79	Cluster 1	4	household		4	3B	3A	
3444424M	Belgium/ULG-11526/2020	EPI_ISL_925470	8	M	Cluster 1		28	child	3	75	72	72	79	Cluster 1	1, 4, 5	school		1, 4, 5	3B	5A	
3448204F	Belgium/ULG-11521/2020	EPI_ISL_925467	8	M	Cluster 1		75	child‡	4		67	67	74	Cluster 1	1, 2, 3, 5	school / household		1, 2, 3, 5	3B	3A	
3450704W	Belgium/ULG-11127/2020	EPI_ISL_925394	8	M	Cluster 1		30	child	5		69			Cluster 1	1, 3, 4, 9	school / household		1, 3, 4, 9	3B		
3015014C	Belgium/ULG-11494/2020	EPI_ISL_925448	39	F	Single 6		21	parent	6	10	12	13	20	Cluster 5	7, 10	school / household		7, 10	1A	4B	
3574033B			6	F			21	child	7		12	13	20	Cluster 5	6, 10, 18, 24,	household		6, 10	1A	4B	
0835014E			55	M			75	parent	8		5	9	16	Cluster 4		school		none	3B	3A	
3122813X			44	F			30	parent†	9	78	82	78	85	Cluster 1	5	household		5	3B		
3334683A			39	M			21	parent	10		12	13	20	Cluster 5	6, 7	school / household		6, 7	4B	1A	
0060599P	Belgium/ULG-11499/2020	EPI_ISL_925453	43	F	Cluster 2		13	parent / teac	11	3	17	17	24	Cluster 2	19, 20, 13, 14	school / household		19, 20, 13, 14	5B	6C	
0252793Z	Belgium/ULG-11514/2020	EPI_ISL_1425567	52	M			66	parent†	12	22	24			Cluster 2	15, 17	household		15, 17	2B	3B	
0254158R	Belgium/ULG-11487/2020	EPI_ISL_925442	35	F	Cluster 2		51	teacher	13	8	10	10	17	Cluster 2	index		creche	index	6A		
0712257N	Belgium/ULG-11503/2020	EPI_ISL_925457	45	F	Cluster 2		45	employee	14	20	17	20		Cluster 2	21, 11, 13, 15	school		21, 11, 13, 15	Bureau		
3185280B	Belgium/ULG-11497/2020	EPI_ISL_925451	37	F	Cluster 2		66	parent / teac	15	11	17	11	18	Cluster 2	12, 17, 14, 21	school		12, 17, 14, 21	2B	3B	
3198375R	Belgium/ULG-11508/2020	EPI_ISL_925461	11	F	Cluster 2		40	child	16	20	19	20	30	Cluster 2	13, 19, 18, 24	school		13	6A		
3532506V	Belgium/ULG-11495/2020	EPI_ISL_925449	8	F	Cluster 2		66	child‡	17		17	11	25	Cluster 2	12, 15, 7, 18,	household		15, 12	2B	3B	
3532943W	Belgium/ULG-11506/2020	EPI_ISL_925459	7	F	Cluster 2		25	child	18	20	18			Cluster2a	24, 25, 16, 19	school		24, 25	2A	6C	
3535356W	Belgium/ULG-11498/2020	EPI_ISL_925452	11	F	Cluster 2		13	child‡	19	3	17	17	24	Cluster 2	11, 20, 16, 17	household		11, 20	6C	5B	
5475157R	Belgium/ULG-11512/2020	EPI_ISL_925463	48	M	Cluster 2		13	parent†	20	20	24	20	27	Cluster 2	11, 19	household		11, 19	6C	5B	
3139292E			49	F			44	employee	21	10	17	18	25	Cluster 2	14, 11, 13, 15	school		14, 11, 13, 15	Bureau		
0621170H			46	M			22	parent	22	15	13	13	20	Cluster2a	23, 25	household		23	2A		
0961887K	Belgium/ULG-11507/2020	EPI_ISL_925460	43	F	Single 5		22	parent	23	6	9			Cluster2a	index	household	son in law	index	2A		
3543568J			7	M			39	child	24		17	18		Cluster2a	18, 25, 16, 17	school		18, 25	2A		
3769892N			7	M			22	child	25	7	9	9	17	Cluster2a	22, 23, 18, 24	school / household		23	2A		
0311161N	Belgium/ULG-11501/2020	EPI_ISL_925455	44	M	Cluster 3		47	parent†	26	19	17	18	25	Cluster 3	28	school / household		28	3A	4A	1B
3611159A	Belgium/ULG-11491/2020	EPI_ISL_925445	36	F	Cluster 3		77	parent†	27	8	10	11	18	Cluster 3a	29	household		29	5B		
4022090L	Belgium/ULG-11500/2020	EPI_ISL_925454	8	F	Cluster 3		47	child‡	28		17	18	25	Cluster 3	26	household		26	3A	4A	1B
4024361V	Belgium/ULG-11488/2020	EPI_ISL_925443	47	M	Cluster 3		77	parent	29	10	10	11	18	Cluster 3a	27	household		27	5B		
0081295S			47	F			42	teacher	30		5	9	12	Cluster 3	11, 13, 14, 15	school		11, 13, 14, 15	4A		
6723472V			38	F			4	parent	31	2	3	2	9	Cluster 3		school		none	4A		
0456683V	Belgium/ULG-11489/2020	EPI_ISL_1425566	40	F	Cluster 4		33	parent	32	4	5	7	14	Cluster 4	35	school / household		35	5B	3B	
3456164V	Belgium/ULG-11493/2020	EPI_ISL_925447	10	F	Cluster 4		7	child	33	11	12	12	19	Cluster 4a	index	household	father	none	5B	2B	
3621255Y	Belgium/ULG-11492/2020	EPI_ISL_925446	7	F	Cluster 4		7	child	34	10	12	12	19	Cluster 4a	index	household	father	none	2B	5B	
0073422J			40	M			33	parent	35	1	-3	-3	4	Cluster 4	32	school / household		32	3B	5B	
0179580Z			54	F			67	teacher	36	8	3	6	20	Cluster 6	index	school	daughter	index	M1		
5323688J	Belgium/ULG-11490/2020	EPI_ISL_925444	37	F	Single 7		61	teacher	37	14	10	12	19	Cluster 6	36, 11, 13, 14	school		36, 11, 13, 14	M1	M3	
0056665C			42	M			19	parent	38	4	5			Cluster 5	index	school	wife	index			
3408828Q			45	F			20	parent	39	16	31	15	18	Cluster 5		school		none			
0474512X	Belgium/ULG-11502/2020	EPI_ISL_925456	47	F	Cluster 4		79	parent	40	12	10	12	22	Cluster 4		school		none			
3066559T			34	F			56	teacher	41	7	9				11, 13, 14, 15, 21, 30, 36, 37, 42			11, 13, 14, 15	5A		
6045553Z			57	F			64	teacher	42		10				11, 13, 14, 15, 21, 30, 36, 37, 41			11, 13, 14, 15	M3A		
0155351A	Belgium/ULG-11515/2020	EPI_ISL_925465	44	F			6	parent											2A	4B	
0637471D			42	M			10	parent											6C	4B	
0546843Y	Belgium/ULG-11513/2020	EPI_ISL_925464	41	F	Single 8		15	parent											6A		

© 2021 Meuris C et al. *JAMA Network Open.*

eTable 7. Posterior Point Estimates for the Parameters of the Generation and Serial Interval Distribution

Table S7. Posterior point estimates for the parameters of the generation and serial interval distribution, and their 95% credible interval (CrI), under different scenarios. SD = standard deviation.

	**Generation interval**		**Serial interval**	
	**Mean (95%CrI)**	**SD (95%CrI)**	**Mean (95%CrI)**	**SD (95%CrI)**
**Scenario 1 (baseline)**	4.67 (3.06 – 6.47)	2.49 (0.98 – 7.17)	4.68 (-4.32 – 14.36)	4.68 (4.08 – 8.19)
**Scenario 2**	4.82 (2.87 – 8.39)	3.37 (1.03 – 12.41)	4.81 (-4.70 – 16.06)	5.20 (4.09 – 13.03)
**Scenario 3**	4.61 (2.75 – 6.76)	3.53 (1.15 – 7.77)	4.60 (-4.96 – 16.24)	5.30 (4.12 – 8.72)
**Scenario 4**	4.74 (3.07 – 6.57)	2.88 (1.03 – 6.43)	4.74 (-4.49 – 15.06)	4.90 (4.09 – 7.56)

© 2021 Meuris C et al. *JAMA Network Open.*
